# Sources and Fluxes of Organic Carbon and Energy to Microorganisms in Global Marine Sediments

**DOI:** 10.3389/fmicb.2022.910694

**Published:** 2022-07-07

**Authors:** James A. Bradley, Sandra Arndt, Jan P. Amend, Ewa Burwicz-Galerne, Douglas E. LaRowe

**Affiliations:** ^1^School of Geography, Queen Mary University of London, London, United Kingdom; ^2^GFZ German Research Center for Geosciences, Potsdam, Germany; ^3^BGeosys, Department of Earth and Environmental Sciences, Université Libre de Bruxelles, Brussels, Belgium; ^4^Department of Earth Sciences, University of Southern California, Los Angeles, CA, United States; ^5^Department of Biological Sciences, University of Southern California, Los Angeles, CA, United States; ^6^MARUM Center for Marine Environmental Sciences, Faculty of Geosciences, University of Bremen, Bremen, Germany

**Keywords:** deep biosphere, organic carbon, bioenergetics, marine sediments, microorganisms

## Abstract

Marine sediments comprise one of the largest microbial habitats and organic carbon sinks on the planet. However, it is unclear how variations in sediment physicochemical properties impact microorganisms on a global scale. Here we investigate patterns in the distribution of microbial cells, organic carbon, and the amounts of power used by microorganisms in global sediments. Our results show that sediment on continental shelves and margins is predominantly anoxic and contains cells whose power utilization decreases with sediment depth and age. Sediment in abyssal zones contains microbes that use low amounts of power on a per cell basis, across large gradients in sediment depth and age. We find that trends in cell abundance, POC storage and degradation, and microbial power utilization are mainly structured by depositional setting and redox conditions, rather than sediment depth and age. We also reveal distinct trends in per-cell power regime across different depositional settings, from maxima of ∼10^–16^ W cell^–1^ in recently deposited shelf sediments to minima of <10^–20^ W cell^–1^ in deeper and ancient sediments. Overall, we demonstrate broad global-scale connections between the depositional setting and redox conditions of global sediment, and the amounts of organic carbon and activity of deep biosphere microorganisms.

## Introduction

Marine sediments comprise one of the largest microbial habitats on the planet (LaRowe et al., 2017; Straume et al., 2019), harboring an estimated 2.9 × 10^29^ microbial cells (Kallmeyer et al., 2012). These organisms are active in sediments that are at least 100 million years old (Morono et al., 2020), driving global biogeochemical cycles (D’Hondt et al., 2019) such as the burial and preservation of organic carbon over geological timescales (Hülse et al., 2018; LaRowe et al., 2020a). The diverse assemblages of organisms in marine sediments (Hoshino et al., 2020) operate over a wide range of energy fluxes (Bradley et al., 2020; Zhao et al., 2021) and physicochemical properties that define the seabed, including water depth, depth within sediments, sediment accumulation rates (Burwicz et al., 2011), mineralogy (Dutkiewicz et al., 2015), amounts and types of organic carbon (LaRowe et al., 2020b), thermal gradients (LaRowe et al., 2017), and pore water electron donors and acceptors (Arndt et al., 2013) including O_2_ and SO_4_^2–^ (Jørgensen and Revsbech, 1989; Jahnke et al., 1990; D’Hondt et al., 2015, 2019; Egger et al., 2018). However, it is unclear how variations in these properties, both vertically and horizontally through space, as well as through time, impact microorganisms on a global scale.

The aim of this study is to quantitatively investigate patterns in the distribution of microbial cells, catabolic zones [i.e., the prevalent redox conditions according to dominant terminal electron acceptors (TEAs)], organic carbon, and catabolic energy, relative to the depth, age, and depositional setting of global subseafloor sediments. Following a previous study that used global datasets, reaction-transport modeling and bio-energetic modeling to quantify the power-utilization of subseafloor microorganisms on a global scale (Bradley et al., 2020), we calculate global-scale changes in sediment physicochemical and biological characteristics according to gradients in sediment depth and age, identifying broad connections between the depositional setting and redox conditions and the amounts of organic carbon and the activity of microbes contained within global subseafloor sediment.

## Materials and Methods

### Experimental Design

Here we use reaction-transport and bioenergetic modeling approaches from Bradley et al. (2020) and LaRowe et al. (2020a) to quantitatively investigate patterns in the distribution of microbial cells, TEAs, organic carbon, and the amounts of power used by microbial cells, relative to the depth, age, and depositional setting of global subseafloor sediments.

We calculate cell-specific power (i.e., catabolic energy utilization per unit time per individual microbial cell), *P* (Watts, W) according to:


(1) $$P=\frac{r\cdot \Delta \,G_{r}}{Y}$$

Where *r* denotes the rate of reaction (g C degraded cm^–3^ s^–1^), Δ*G*_*r*_ represents the Gibbs energy of the reaction (J g^–1^ C), and *Y* (cells cm^–3^) is the number of microbial cells carrying out the reaction. The modeling framework is implemented on a 0.25° × 0.25° resolution global grid, using global datasets and models described below and summarized in Supplementary Table 1.

### Reaction-Transport Model

We use a one-dimensional reaction-transport model (RTM) (implemented in MATLAB R2022a) to calculate the distribution and degradation rate of particulate organic carbon (POC) throughout the Quaternary Period, following the approach described in Bradley et al. (2020) and LaRowe et al. (2020a). Quaternary sediments were divided into three layers: bioturbated Holocene [i.e., the top 10 centimeters below the sea floor (cmbsf)], non-bioturbated Holocene (10 cmbsf to sediments that are 11,700 years old) and Pleistocene (11,700-years-old to 2.59-million-years-old sediments). Sediment mixing was assumed to be constant over the bioturbated layer and non-existent immediately below it. Similar to Wallmann et al. (2012), constant boundary conditions and parameters are used to characterize the Holocene and Pleistocene depositional environments.

The one-dimensional conservation equation for POC in porous media is given by Berner (1980) and Boudreau (1997):


(2) $$\frac{\partial \left(1-\Phi \right)P\,O\,C}{\partial t}=\frac{\partial }{\partial z}\,\left(D_{b}\,\left(1-\Phi \right)\,\frac{\partial P\,O\,C}{\partial z}\right)\;-\frac{\partial \left(1-\Phi \right)\omega \,P\,O\,C}{\partial z}+\left(1-\Phi \right)\,R_{P\,O\,C}$$

Where *POC* is the concentration of POC (g C cm^–3^ dry sediment); *t* refers to time (years); *D*_*b*_ (cm^2^ year^–1^) stands for the bioturbation coefficient; ω (cm year^–1^) represents the sedimentation rate and *R*_*POC*_ denotes the rate of organic matter degradation (g C cm^–3^ year^–1^). The porosity, Φ, of marine sediments in the shelf, margin, and abyss domains was calculated as a function of depth, *z* (m), assuming steady-state compaction, according to Athy (1930):


(3) $${\text{Φ}}_{z}={\text{Φ}}_{0}\cdot e^{-c_{0}\cdot z}$$

where *Φ_0_* denotes the porosity at the sediment-water interface (SWI) and *c*_0_ (m^–1^) stands for the compaction length scale, which characterizes how a given sediment type will compact under its own weight.

The rate of organic matter degradation, *R*_*POC*_, was described using a reactive continuum model (RCM). The RCM assumes a continuous, yet dynamic, distribution of organic compounds comprising a range of reactivities and reproducing the often-observed decrease in apparent POC reactivity with increasing sediment depth (and thus age) (Boudreau and Ruddick, 1991). Within the RCM, *R*_*POC*_ is given by:


(4) $$R_{P\,O\,C}=-\int_{0}^{\infty }k\cdot o\,m\,\left(k,t\right)\,d\,k$$

Where *om(k,t)* represents a probability density function that determines the concentration of organic matter having a degradability between *k* and *k+dk* at time *t*, with *k* being analogous to a reaction rate constant. The initial distribution of organic compounds [*om(k,0)*] cannot be inferred by observations and may take different mathematical forms. We use a gamma function (Aris, 1968; Ho and Aris, 1987; Boudreau and Ruddick, 1991; LaRowe et al., 2020a), assuming first order degradation kinetics, whereby the initial (*t* = 0) distribution of *om* over *k* is given by:


(5) $$o\,m\,\left(k,0\right)=\frac{P\,O\,C_{0}\cdot a^{\nu }\cdot k^{\nu -1}\cdot e^{-a\cdot k}}{\text{Γ}\,\left(\nu \right)}$$

where *POC*_0_ is the initial organic matter content (at the SWI), Γ is the gamma function, *a* (years) is the average lifetime of the reactive components of the POC and ν is a dimensionless parameter determining the shape of the distribution near *k* = 0. Assuming steady-state conditions (∂*POC/*∂*t = 0*) and a known organic C content at the sediment water interface, *POC*_0_, the change in the POC concentration as a function of depth, *POC(z)*, is given by Boudreau and Ruddick (1991):


(6) $$P\,O\,C\,\left(z\right)=P\,O\,C_{0}\cdot {\left(\frac{a}{a+a\,g\,e\,\left(z\right)}\right)}^{\nu }$$

where *age(z)* refers to the age of the sediment layer at depth *z*. Further details on the reaction transport model and the calculation of the POC budget are provided in the Supplementary Material.

### Parameters and Forcings

**FIGURE 1:**
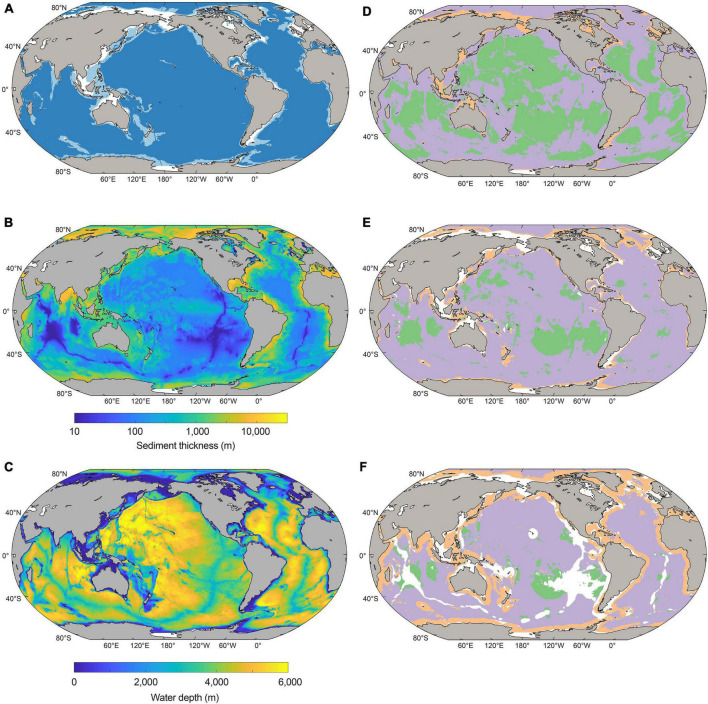
**(A)** Geographic distribution of the shelf (white), margin (light blue), and abyss (dark blue) sediment domains considered in this study. **(B)** Sediment thickness (meters). **(C)** Water depth (meters). **(D–F)** Global maps depicting the major catabolic pathway of particulate organic carbon (POC) degradation in 10,000-year-old sediments **(D)**, 100,000-year-old sediments **(E)**, and 1-million-year-old sediments **(F)**. Green shading represents oxic sediments, lilac denotes sulfate-reducing sediments, orange signifies methanogenic sediments, and white corresponds to areas where there are no sediments of the specified age.

The concentration of POC at the sediment water interface (*POC*_0_) during the Holocene and Pleistocene, and sedimentation rates, ω, are constrained according to Wallmann et al. (2012) who used data from Seiter et al. (2004), Romankevich et al. (2009) and an algorithm that correlates water depth and sedimentation rate (Burwicz et al., 2011). The bioturbation coefficient is calculated as a function of water depth based on a compilation of empirical data collected by Middelburg et al. (1997). Its values range spatially from 0.59 to 27 cm^2^ year^–1^, decreasing in magnitude as water depth increases. It is constant throughout the depth of the Holocene bioturbated zone and immediately drops to zero beneath it. Values of *Φ_0_* and *c*_0_ are chosen to describe the shelf, margin and abyss based on sediments that are representative of these domains (Hantschel and Kauerauf, 2009; see Supplementary Table 1.1). We partitioned the ocean floor into shelf, margin and abyss domains to specify values for some of the model parameters (Supplementary Table 1.1). The locations of the continental margin boundaries are adopted from Vion and Menot (2009) and inform the definition of each domain: shelf environments roughly correspond to water depths <200 m, with the exception of the Antarctic region where shelf area corresponds to water depths <500 m, areas deeper than ∼3500 m and associated with oceanic crust environments are taken to be abyssal plain; and ocean floor covered by 500–3500 m water are referred to as margins (Figure 1A). We choose a constant ν parameter of 0.125 for all three sediment domains, which is characteristic of fresh organic matter and based on observations that the ν parameter values do not vary much between sites (Boudreau and Ruddick, 1991; Arndt et al., 2013). We link values of the *a* parameter with sedimentation rates, based on global observations (Boudreau and Ruddick, 1991; Arndt et al., 2013). This approach accounts for order-of-magnitude changes in *a* due to factors that control organic matter transit times from its source to deposition. These parameters thus reflect typically observed RCM parameter variability across various depositional environments.

### Global Reaction Network, Electron Acceptors and Catabolic Zones

We use the oxygen penetration depths and sulfate–methane transition (SMT) depths established in Bradley et al. (2020). Briefly, oxygen penetration depths in global marine sediments are estimated based on a compilation of global datasets of oxygen concentrations in marine sediments combined with previous modeling (D’Hondt et al., 2015; Supplementary Table 1.2). We use data from Egger et al. (2018) to map the global SMT depth onto our 0.25° × 0.25° global grid. We assign all sediments shallower than the maximum O_2_ penetration depth as oxic. We designate the sulfate-reduction zone as the horizon between the maximum O_2_ penetration depth and the depth of the SMT. We prescribe POC degraded beneath the SMT to methanogenesis (Bradley et al., 2020; Supplementary Table 2). We use acetate (CH_3_COO^–^) as a proxy for POC (Bradley et al., 2020).

### Gibbs Energy Calculations

The amount of energy available from the oxidation of organic matter by aerobic, sulfate-reducing and methanogenic pathways (Supplementary Table 2) is calculated based on the Gibbs energy function:


(7) $$\text{Δ}\,G_{r}=\text{Δ}\,G_{r}^{0}+R\,T\,\text{ln}\,Q_{r}$$

where Δ*G*_*r*_^0^ and *Q*_*r*_ refer to the standard molal Gibbs energy and the reaction quotient of the indicated reaction, respectively, *R* represents the gas constant, and *T* denotes temperature in Kelvin. Values of Δ*G*_*r*_^0^ were calculated using the revised-HKF equations of state (Helgeson et al., 1981; Tanger and Helgeson, 1988; Shock et al., 1992), the SUPCRT92 software package (Johnson et al., 1992), and thermodynamic data taken from Shock and Helgeson (1988), Shock and Helgeson (1990), Shock et al. (1989), Sverjensky et al. (1997), Schulte et al. (2001). Individual values of *Q*_*r*_ are calculated for each reaction using:


(8) $$Q_{r}=\prod \,a_{i}^{v_{i}}$$

where *a*_*i*_ stands for the activity of the *i*th species and *v*_*i*_ corresponds to the stoichiometric coefficient of the *i*th species in the reaction of interest. Activity of the *i*th species is calculated according to:


(9) $$a_{i}=m_{i}\,\gamma_{i}$$

where *m*_*i*_ and γ_*i*_ denote the molality and individual activity coefficient of the *i*th species, respectively. Values of γ_*i*_ are computed as a function of temperature and ionic strength using an extended version of the Debye-Hückel equation (Helgeson, 1969).

We have selected specific sets of representative conditions that are used to calculate Gibbs energies of each reaction (Supplementary Table 3). We define reactions at 5°C and 100 bars of pressure. For aerobic heterotrophy, we base our calculations on chemical data from oxic South Pacific Gyre sediments (D’Hondt et al., 2015). For sulfate-reducing and methanogenic sediments, we use chemical data from anoxic Limfjorden and Peru Margin sediments (D’Hondt et al., 2004; Jørgensen and Parkes, 2010).

### Cell Abundance

Global subseafloor cell abundance was estimated based on a power law formulation from Kallmeyer et al. (2012):


(10) $$Y=b\cdot z^{m}$$

Where *Y* is cell abundance (cells cm^–3^), *z* denotes the depth below the seafloor (m), and *b* and *m* are parameters based on mean sedimentation rate and distance from landmasses greater than 10^5^ km^2^. *b* and *m* were interpolated onto our 0.25° × 0.25° global grid based on data provided in Kallmeyer et al. (2012). Cell concentration in the upper-most bioturbated layer (*Y*_*zbio*_, 0–10 cm) was uniformly described, following (Kallmeyer et al., 2012), as:


(11) $$Y_{z\,b\,i\,o}=b\cdot 0.1^{m}$$

Cell-specific catabolic rates are calculated by dividing total power by total cell numbers, thus assuming that all cells are equally performing the catabolic metabolism associated with that particular catabolic zone (i.e., aerobic heterotrophy in the oxic zone, sulfate reduction in the sulfate-reducing zone, methanogenesis in the methanogenic zone, as indicated in Supplementary Table 3).

### Global Totals

We quantified total subseafloor sedimentary volume, electron acceptors and cell abundance from the SWI to the basement. We calculated POC stored and degraded, as well as power utilized, from the SWI to the deepest quaternary-aged sediments by integrating across specific sediment depth and age horizons.

## Results and Discussion

### Depositional Settings and Distribution of Terminal Electron Acceptors in Subseafloor Sediments

The geographical distribution of major sediment domains (shelf, margin, and abyssal zones) is shown in Figure 1A. Shelf settings underlie 6.3% of the seafloor, broadly corresponding to areas of shallow water (<200 m water depth) including the Siberian Shelf, the Sunda and Sahul shelves off Southeast Asia and Australia, the North Sea, the Atlantic coast of South America, and the southern-most parts of the Southern Ocean. Margin settings generally surround the shelf, and make up 10.8% of the seafloor. Abyssal settings constitute the largest marine domain (82.9% of the seafloor): this zone accounts for most of the seafloor area underlying major ocean basins and also includes the mid-ocean ridges.

As per our previous study (Bradley et al., 2020), we designate all sediments shallower than the maximum O_2_ penetration depth as oxic. We then define the sulfate-reduction zone as the horizon between the maximum O_2_ penetration depth and the depth of the SMT. Finally, we define the methanogenic zone as the sediments deeper than the SMT. Our framework does not imply that the oxic zone is devoid of sulfate (or other TEAs), but rather that O_2_ is an available TEA in this zone.

The redox zones of global sediment at three distinct sediment age horizons representing different geological timescales are shown in Figures 1D–F: sediment deposited (Figure 1D) 10,000 years ago, (Figure 1E) 100,000 years ago, and (Figure 1F) 1 million years ago. In 10,000-year-old sediment, oxygen and sulfate are widespread in abyssal zones, whilst margin sediments are predominantly methanogenic. Oxygen is less prevalent in deeper and more ancient sedimentary layers (corresponding to sediment that was deposited 100,000 years ago) (Figure 1E) than younger (and thus shallower) sediments. The proportion of abyssal sediments that are sulfate-reducing increases in the 100,000-year-old age horizon relative to the younger deposits above. Methanogenic sediments are also more widespread in 100,000-year-old sediments than in younger and shallower sediment – prevalent mostly on shelves and margins, particularly in northern high latitudes. Regions shown in white do not bear sediment that is 100,000 years old; these regions correspond largely to sediment close to continental shelves that were either exposed due to sea-level drop or covered by glaciers and ice sheets during the Pleistocene. Ancient sediments deposited 1 million years ago are indicated by shading in Figure 1F, whilst white corresponds to areas of the seafloor which do not bear 1 million-year-old sediment (exposed or ice-covered shelf regions, and spreading centers). These ancient sediment layers are mostly anoxic, with the exception of the deepest abyssal zones (Figure 1F) in the south Pacific, north Atlantic, mid-Indian, and Wharton basins, where oxygen penetrates tens of meters below the seafloor (D’Hondt et al., 2009,D’Hondt et al., 2011, 2015; Roy et al., 2012; Orcutt et al., 2013; Vuillemin et al., 2020a). The vast majority of abyssal sediment at the 1-million-year-old horizon is sulfate-reducing, whilst shelf and margin sediment of this age is predominantly methanogenic, especially in areas within ∼1,000 to 2,000 km distance of coastlines.

The patterns shown in Figure 1 are partly explained by the fact that horizons of equal sediment age do not necessarily correspond to equal sediment depth below the seafloor. Whilst it is true that more recently deposited sediment broadly corresponds to shallower layers, and similarly that ancient sediment generally corresponds to deeper layers, there are enormous spatial heterogeneities in the actual depth below the seafloor of equivalent sediment ages. This arises from considerable differences in sedimentation (and thus burial) rate across space and time. For example, a reference depth of ∼10 meters below the sea floor (mbsf) represents ∼11,000 years of burial in shelf sediments in Aarhus Bay, Denmark (Langerhuus et al., 2012), yet millions of years of burial in abyssal zones, for example in the South Pacific Gyre (D’Hondt et al., 2015).

**FIGURE 2:**
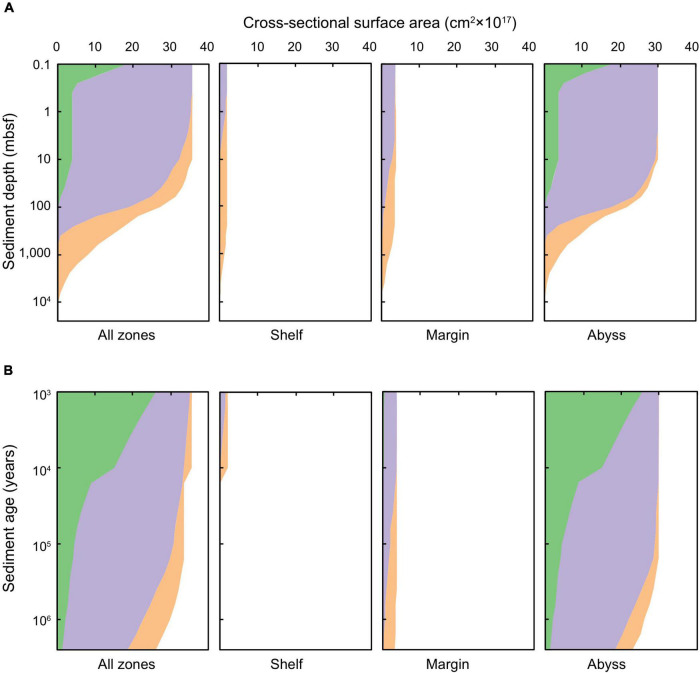
Cross-sectional surface area of global marine sediments according to **(A)** sediment depth (0.1 to ∼10,000 mbsf) and **(B)** sediment age (1,000 years to 2.59 million years). Green shading represents oxic sediments, lilac denotes sulfate-reducing sediments, and orange signifies methanogenic sediments.

The cross-sectional surface areas of global Quaternary-age marine sediment slices as characterized by redox-states and depositional settings are shown as a function of sediment depth (0.1 to ∼10,000 mbsf) and age (1,000 years to 2.59 million years) in Figure 2. The total cross-sectional area of marine sediments at specific depths or ages declines with increasing depth and age, respectively, because there is overall less sediment in deeper and older settings globally than there is at the SWI. Oxic sediments are most-prevalent near the SWI. Sulfate-reducing sediments are prevalent at intermediary depths and ages, and methanogenic sediments are most prevalent in deeper and older horizons. We calculate that there is oxygen in 2.7% of Quaternary-age sediments by volume, and in 1.1% of total global subseafloor sediments by volume. These values agree well with (Estes et al., 2019). Oxygen is present mostly in shallow (<1 mbsf) and recently deposited (<10,000 years) sediments in abyssal zones. We calculate that 13.4% of total global sediment volume is sulfate-reducing, predominantly in sediment depths ranging between 0.3 and 100 mbsf, and sediment ages from 10,000 to 1 million years. The overwhelming majority of global sediment volume is methanogenic (85.5% of total global sediments). Methanogenic sediments are generally found in deeper sedimentary layers of the continental shelves, which can be hundreds of meters to several kilometers thick (Egger et al., 2018; Figure 1B).

Broadly, shelf sediments are anoxic from very close to the SWI (i.e., the deepest bioturbated layer) to the basement. These sediments are primarily sulfate-reducing in the shallowest depths (<1 mbsf) and youngest ages (<1,000 years), and transition to methanogenic with increasing depth beneath the seafloor (up to thousands of meters) (Figure 2). There is a sharp decline in the total cross-sectional surface area of shelf sediments at the Holocene-Pleistocene boundary (Figure 2B), which arises due to past Pleistocene sea-level drop and glaciations covering vast regions of the ocean with ice (in particular, regions that are now continental shelf settings). Margin sediments comprise roughly twice the areal extent of shelf sediments, and exhibit similar redox characteristics to the shelf setting. In the shallowest and youngest horizons (<10 mbsf and <10,000 years, respectively), sediments are predominantly anoxic and sulfate bearing. Within these horizons there are only a few dispersed patches of oxic and methanogenic sediment. At sediment depths greater than 10 mbsf or older than 10,000 years, sediments are primarily methanogenic – and are increasingly so with sediment depths >1,000 mbsf and ages >1 million years.

Abyssal sediments are primarily oxic in the upper-most (<0.1 mbsf) and youngest (buried in the last ∼10,000 years) zones, in broad agreement with previous empirical (D’Hondt et al., 2015) and modeling studies (Archer et al., 2002). Oxygen persists over million-year timescales and to ∼100 mbsf in ∼5% of abyssal marine sediments by cross-sectional area. Nevertheless, the majority of deep and ancient abyssal sediments are anoxic: sulfate-reduction and methanogenesis being prevalent in sediments in the deepest (>300 mbsf) and oldest (>1 million years) abyssal zones. This result aligns with observations of anoxic abyssal sediments occurring, in particular, outside of the ultra-oligotrophic regions of the ocean, and beyond the first tens of centimeters to meters beneath the seafloor (Murray and Varis, 1980; Jahnke et al., 1982; D’Hondt et al., 2009; Zhao et al., 2020, 2021; Versteegh et al., 2021; Møller et al., 2022).

Calculations of heat-flow and thermal conductivity of marine sediments suggest that temperatures rise significantly in deeper and thicker sedimentary layers: up to a quarter of deep marine sediments (by volume) are above 80°C (taken as a major thermal barrier for diffusion-dominated subsurface life) (LaRowe et al., 2017), potentially leaving the deepest methanogenic sediments largely inhabitable to subseafloor life.

### Distribution of Cells in Oxic and Anoxic Sediments

**FIGURE 3:**
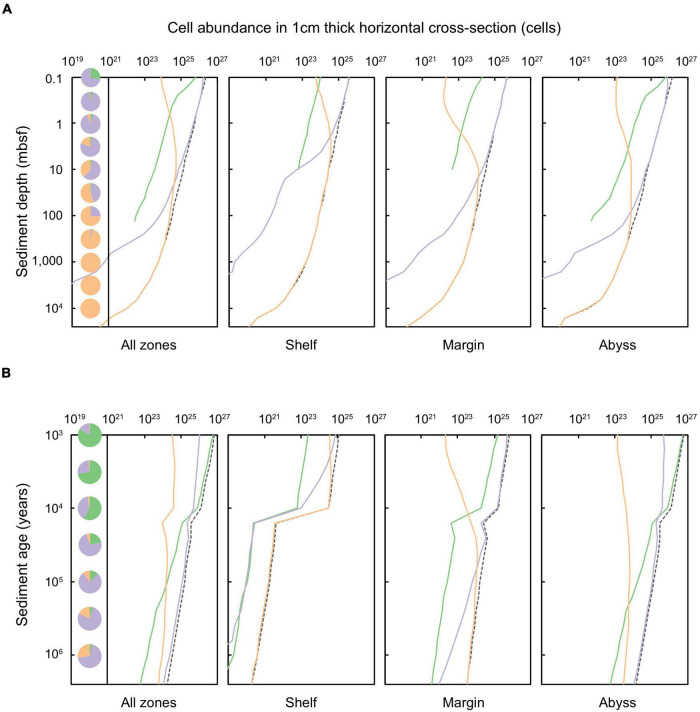
Total cells in a 1 cm thick layer of marine sediments deposited up to 2.59 million years ago, according to equal **(A)** sediment depth (shown for sediments at 0.1 to ∼10,000 mbsf) and **(B)** sediment age (1,000 years to 2.59 million years) for shelf, margin, abyss, and total global sediments. The dashed black line represents the total cells at specific depth or age layers. Solid lines indicate the cell abundance in specific sediment catabolic zones in which cells are situated: green represents oxic sediments, lilac denotes sulfate-reducing sediments, and orange signifies methanogenic sediments. Pie charts indicate the proportion of cells of that layer that are stored within oxic, sulfate-reducing, and methanogenic sediments.

There are ∼2.9 × 10^29^ cells contained in marine sediments globally (Kallmeyer et al., 2012), compared to ∼2–6 × 10^29^ cells contained in the continental subsurface (Magnabosco et al., 2018). In global subseafloor sediments, we calculate that 1.8% of cells are situated in oxic zones, whilst 16.4% are found in sulfate-reducing zones, and 81.8% are contained in methanogenic zones. For Quaternary-age sediments these proportions are: 3.0, 38.0, and 59.0%, respectively (Bradley et al., 2020). There is a broad exponential decline in cell abundance with increasing depth and age in global marine sediments (Figure 3), which is consistent with what is observed in empirical studies (Kallmeyer et al., 2012; D’Hondt et al., 2015; Kirkpatrick et al., 2019). This global trend is also reflected in the specific sediment domains: shelf, margin, and abyss. Notably, cell abundance decreases sharply in shelf sediments at the ∼11,700 year old horizon (the Pleistocene-Holocene boundary), reflecting the decrease in the total volume of sediment of this age due to Pleistocene sea-level drop and glaciation (Figure 2). The models for cell distribution with sediment depth [from Kallmeyer et al. (2012)], as well as with sediment age and catabolic zone (this study) do not account for any zones where there may be locally higher abundances of cells at greater sediment depths, for example at North Pond ridge flank – where rapid basement water circulation and concomitant diffusion upward leads to the presence of oxygen beneath an anoxic sediment zone (Orcutt et al., 2013).

Cells within oxic sediment are restricted almost entirely to the shallowest depths. Slightly more than a quarter (27.5%) of cells at 0.1 mbsf are in oxic sediments (Figure 3A); these are mostly in the abyssal domain (Figure 3). Beneath 0.1 mbsf, the proportion of cells in oxic sediment (abyssal and global) declines, and thereafter microbial cells are predominantly situated in anoxic sediment (Figure 3A). Cells in sulfate-reducing sediment dominate the subseafloor globally from 0.1 to 30 mbsf, especially in margin and abyssal zones. In shelf settings, a greater proportion of cells are in methanogenic sediment. Globally, at 100 mbsf and deeper, almost all microbial cells are present in methanogenic sediments.

The abundances of microbial cells within sediments of equal age (i.e., time since deposition and burial) are shown in Figure 3B, and are further distinguished according to redox setting. Almost all microbial cells in global shelf (97.9%) and margin (76.5%) zones are contained in anoxic sediments. Most microbial cells contained in shelf sediments are situated in sulfate-reducing conditions for sediment deposited up to ∼2,000 years ago. Beyond this, in older sediments, methanogenesis dominates. In margin zones, microbial cells in sulfate-reducing sediments persist to ∼50,000 years since burial, beyond which cells are primarily situated in methanogenic sediment.

The distribution of subseafloor-sediment microbes is strongly correlated with mean sedimentation rate and distance from land. For example, sediments in shelf zones have significantly higher cell concentrations per unit volume (10^6^–10^10^ cells cm^–3^) than abyssal zones (10^2^–10^5^ cells cm^–3^) (Kallmeyer et al., 2012). We find that despite harboring low concentrations of cells per unit volume, abyssal sediments contain the majority of global sediment-dwelling microbial cells (40.2%), whilst 29.3% of sediment-dwelling cells are contained in shelf sediments and 30.5% of sediment-dwelling cells are contained in margin sediments. Whereas shelf and margin sediments rapidly become anoxic within just millimeters to centimeters beneath the sediment-water interface, most cells in abyssal sediment are exposed to oxygen throughout at least the first 10,000 years of burial, and in some cases, for up to millions of years (Fischer et al., 2009; D’Hondt et al., 2015; Morono et al., 2020). Abyssal sediments are buried extremely slowly (in some cases by ∼1 m of sediment every million years), and the degradation rate of organic matter (coupled to the consumption of oxygen) is also very slow (D’Hondt et al., 2015). Oxygen therefore persists in abyssal zones over very long timescales, and accordingly, 3–5% of cells in sediment older than 1 million years are in oxic conditions. Nevertheless, despite the presence of oxygen in abyssal zones over long timescales as observed by D’Hondt et al. (2015) and others, we find that the vast majority of global marine sediment volume (98.9%) is anoxic, and in turn, that the vast majority of sediment-dwelling microbial cells (98.2%) subsist under anoxic conditions. This result is further underscored by empirical observations of anoxic abyssal sediments outside of the ultra-oligotrophic regions of the ocean and at greater depths beneath the seafloor (Murray and Varis, 1980; Jahnke et al., 1982; D’Hondt et al., 2009; Zhao et al., 2020, 2021; Versteegh et al., 2021; Møller et al., 2022).

### Particulate Organic Carbon

**FIGURE 4:**
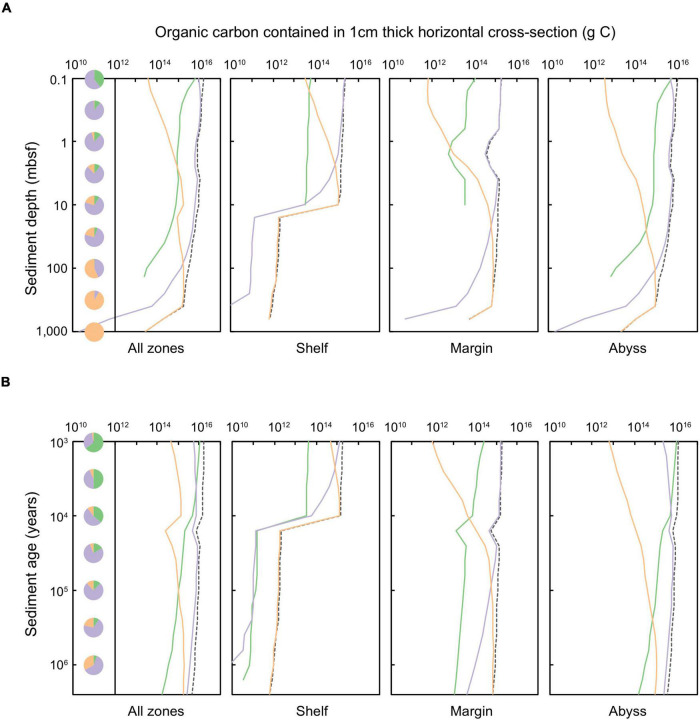
Total organic carbon stored in a 1 cm thick layer of global marine sediments deposited up to 2.59 million years ago, according to equal **(A)** sediment depth (shown for sediments at 0.1 to ∼10,000 mbsf) and **(B)** sediment age (1,000 years to 2.59 million years). The dashed black line represents the total organic carbon contained in specific depth or age layers. Shading of solid lines indicates the organic carbon contained in sediments of a specific catabolic zone: green shading represents oxic sediments, lilac denotes sulfate-reducing sediments, and orange signifies methanogenic sediments. Pie charts indicate the proportion of total POC that is stored within all oxic, sulfate-reducing, and methanogenic sediments of a specific **(A)** depth or **(B)** age horizon.

**FIGURE 5:**
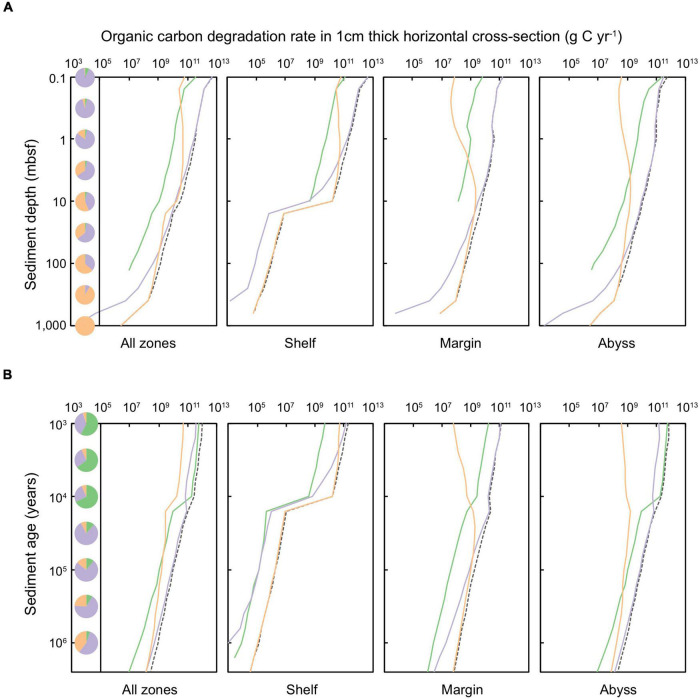
Organic carbon degradation rate in a 1 cm thick layer of global marine sediments deposited up to 2.59 million years ago, according to equal **(A)** sediment depth (shown for sediments at 0.1 to ∼10,000 mbsf) and **(B)** sediment age (1,000 years to 2.59 million years). The dashed black line represents the total organic carbon degradation rate in specific depth or age layers. Shading of solid lines indicates the organic carbon degraded in sediments of a specific redox-state: green shading represents oxic sediments, lilac denotes sulfate-reducing sediments, and orange signifies methanogenic sediments. Pie charts indicate the proportion of total POC that is degraded in oxic, sulfate-reducing, and methanogenic settings for a specific **(A)** depth or **(B)** age horizon.

We quantify the POC stored (Figure 4) and degraded (Figure 5) in Quaternary-age marine sediments according to horizons of equal sediment depth (Figures 4A, 5A) and sediment age (Figures 4B, 5B). The amount of POC stored within sediment layers decreases with depth and time since burial. Patterns in the distribution of POC – both across depositional settings and according to redox zones – broadly resemble patterns observed in the distribution of microbial cells.

Despite generally lower POC concentrations in abyssal sediments compared to POC-rich shelf and margin settings (LaRowe et al., 2020a), most of the total POC in global Quaternary-age sediment (1.46 × 10^20^ g C) is stored in abyssal (1.01 × 10^20^ g C, 69.2% of total Quaternary sediment POC) and margin zones (4.30 × 10^19^ g C, 29.4% of total Quaternary sediment POC). Relatively little of the POC stored in global Quaternary-age sediments is on the continental shelf (2.03 × 10^18^ g C, 1.4% of total Quaternary sediment POC), although this estimate is conservative as it does not include particularly POC-rich coastal systems such as deltas and fans.

A minor fraction (∼4.6%) of Quaternary-age sediment POC is stored in oxic settings (Bradley et al., 2020), aligning with previous empirical measurements and modeling efforts (Estes et al., 2019; Bradley et al., 2020). POC in oxic sediment is located primarily within the upper-most (<1 mbsf) and youngest (<10,000 years) abyssal zones. Aerobic heterotrophy is responsible for only 6.9% of POC degradation in global Quaternary sediments – limited to just the first ∼3,000 years following deposition (Figure 5B). The vast majority of Quaternary-age sediment POC is contained in anoxic settings: predominantly POC is contained in sulfate-bearing sediments in the uppermost 100 m of all depositional environments (shelf, margin, and abyssal zones), and methanogenic sediments in shelf zones deeper than ∼3 mbsf and margin sediments deeper than ∼30 mbsf. Following the distribution of POC, anoxic pathways are also responsible for the majority of Quaternary-age POC degradation (93.1%) (Figure 5), in agreement with a previous reaction-transport based modeling study (Thullner et al., 2009). Sulfate-reduction (which, we estimate, is responsible for 64.5% of global Quaternary POC degradation) dominates POC degradation in shallow settings (<10 mbsf) whilst most POC in sediments buried deeper than 10 mbsf is degraded via methanogenesis (28.6% of global Quaternary POC degradation) (Figure 5).

Due to necessary simplifications to the modeling framework, it is likely that, in some cases, we misattribute some organic carbon degradation between oxic, sulfate-reducing and methanogenic pathways, as well as between pathways that are not explicitly included in this study, such as fermentation (Lever, 2012; Vuillemin et al., 2020b). However, we expect that these simplifications lead to minor deviations from the broad scale patterns in sedimentary setting, depth, and time reported here (see discussion of limitations and uncertainty in Section “Limitations and Uncertainty of Model Predictions”).

Field observations have revealed that rates of POC degradation span over eight orders of magnitude (Middelburg, 1989; LaRowe et al., 2020a). Our results suggest that despite variation in POC degradation across age, depth, depositional zone, and the redox-state of sediment, degradation rates largely reflect the distribution of POC stored, such that: where there is a higher concentration of POC at a particular depth, age, or depositional setting, it is generally degraded at a faster rate.

### Catabolic Power Utilization

**FIGURE 6:**
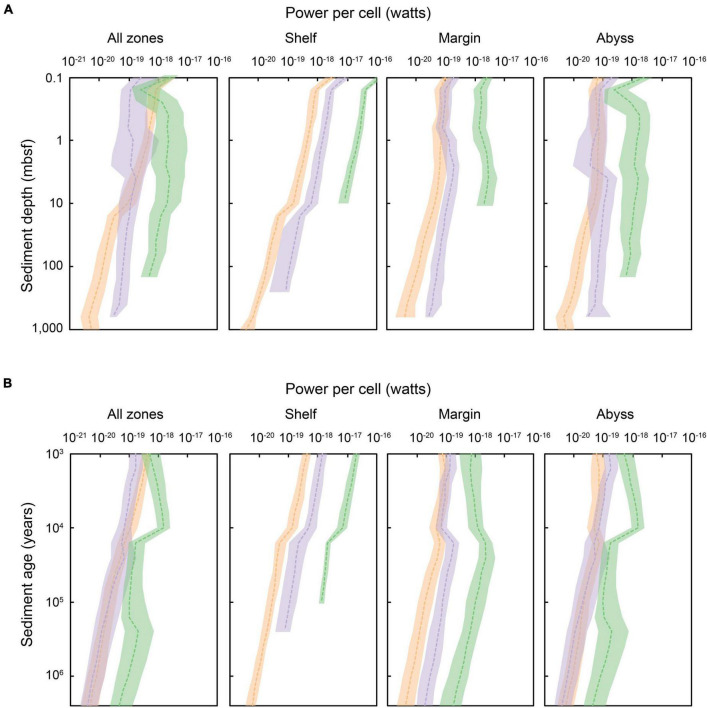
The energy flux (i.e., power) per cell in global marine sediments, according to equal **(A)** sediment depth (shown for sediments at 0.1 to ∼10,000 mbsf) and **(B)** sediment age (1,000 years to 2.59 million years). Shading indicates the redox-state of the sediment in which cells are situated: green shading represents oxic zones, lilac denotes sulfate-reducing zones, and orange signifies methanogenic zones. The dashed colored lines represent the median power per cell within a specific horizon, depositional environment, and redox setting. Power per cell is spatially variable for a given sediment depth or age layer. The shaded area indicates the upper and lower quartiles of power per cell for a specific depth or age horizon.

The rate of energy used per individual microbial cell (i.e., power per cell; W cell^–1^) in sediments varies by orders of magnitude across space and time, mostly within the range of ∼10^–20^ to 10^–16^ W cell^–1^. This range is in line with previous estimates of the power utilization of microorganisms in marine sediments (Bradley et al., 2020; Zhao et al., 2021), and is several orders of magnitude less than the power use of chemolithoautotrophs in soils (10^–12^ to 10^–17^ W cell^–1^) (Bay et al., 2021), and the lowest maintenance powers estimated for laboratory-grown cultures of aerobic heterotrophs [which average 1.9 × 10^–15^ W cell^–1^, and range between 10^–12^ and 10^–17^ W cell^–1^ (Tijhuis et al., 1993; DeLong et al., 2010; Marschall et al., 2010; Kempes et al., 2017)]. Figure 6 shows how the power utilization per cell varies with depth, age, depositional setting, and catabolic zone in global marine sediments. Cells in shallow (>0.1 mbsf) and/or recently deposited (>1,000 years) sediment use around 10^–19^ to 10^–18^ W cell^–1^. Generally, the power used per cell declines with increasing sediment depth and sediment age, at a rate of one to two orders of magnitude decrease in power per ∼1,000 m of depth beneath the seafloor or per 1 million years of burial. The cells using the lowest amounts of power (∼2 × 10^–21^ to 10^–19^ W cell^–1^) are found in deep (∼1,000 mbsf) and ancient (>1 million years) anoxic marine sediment.

There is a clear delineation of microbial power utilization according to catabolic zones. In general, aerobic heterotrophs (situated in oxic sediments) use the highest amounts of power per cell, followed by sulfate-reducers (∼1 order of magnitude lower than microorganisms in oxic sediments), and then methanogens (∼1 order of magnitude lower than microorganisms in sulfate-reducing sediments). This pattern is reflected globally in results presented in Bradley et al. (2020). Geochemical transition zones provide greater amounts of power for microbes carrying out certain metabolisms, including the SMT zone for methane oxidizers and sulfate-reducing bacteria (Boetius et al., 2000; Parkes et al., 2005), the oxic–anoxic transition zone for nitrifiers (Zhao et al., 2019), and the nitrate–ammonium transition zone for anammox bacteria (Zhao et al., 2020). The power landscape of these important transition zones is not explicitly captured by our modeling framework and associated results. Likewise, cryptic cycling (Holmkvist et al., 2011; Garber et al., 2021), metabolic versatility (Dombrowski et al., 2018), and other metabolic pathways (Zhao et al., 2019) are not considered here (see Section “Limitations and Uncertainty of Model Predictions”).

We find distinct trends in cell-specific power regime across different depositional settings (Figure 6). Microorganisms in shallow and recently deposited shelf sediments subsist at the highest power per cell (∼10^–16^ W cell^–1^) (Bradley et al., 2020). Shelf sediments are typically characterized by higher cell abundance near the sediment-water interface, and steep declines in cell abundance with increasing depth below the seafloor (and thus sediment age), which may be associated with high rates of cell mortality (Langerhuus et al., 2012; Lomstein et al., 2012; Braun et al., 2017). We observe a concurrent steep decline in per-cell power utilization with increasing depth and age in shelf sediments, and suggest a link between the rapid declines in cell abundance observed in coastal sediments and extreme power limitation. We find that microbial cells in shelf sediment that is buried >1,000 mbsf or that is >1 million years old subsist at <10^–20^ W cell^–1^, nearing the lowest power known to support any subseafloor-sediment microbes (LaRowe and Amend, 2015; Bradley et al., 2020). Abyssal zones have relatively more consistent availability of power on a per cell basis over gradients in sediment depth and burial history. In general, microbial cells in abyssal sediments are sustained at very low cell concentrations (10^2^–10^5^ cells cm^–3^) (Kallmeyer et al., 2012; D’Hondt et al., 2015) and utilize very low amounts of power (LaRowe and Amend, 2015), but are shown to persist for millions of years (Morono et al., 2020) with extremely low maintenance power requirements (Bradley et al., 2018a) and mortality rates (Bradley et al., 2018b,^2019^). The apparent survival of abyssal microbial cells over extraordinary timescales may be linked to a low but relatively constant power availability.

### Limitations and Uncertainty of Model Predictions

We use a combination of empirical data and modeling approaches (reaction-transport, and bioenergetic/thermodyn amic) to derive first-order estimates of the distribution of cells, TEAs, POC and catabolic energy availability in global marine sediments. The results are subject to uncertainties introduced through the model design and parameterization, as well as simplifications and unknowns.

Importantly, the modeling framework and results presented here are appropriate for broad regions of the seafloor but do not capture variation in biogeochemical dynamics arising from certain atypical subseafloor phenomena and features such as thermal anomalies (Heuer et al., 2020), and hydrothermal fluid circulation through seamounts and ridge flanks (Wheat et al., 2019; Price et al., 2022). Moreover, the properties and reactivity of sedimentary POC are known to vary greatly across depositional environments as well as in space and time – driven both by differences in POC chemical composition and structure as well as bio-physicochemical properties of the environment (LaRowe et al., 2020b). Modeled POC concentrations and degradation rates depend primarily on the variability in the concentration and reactivity of POC deposited onto the seafloor and its evolution during burial, which are accounted for within the model by parameters representing the average lifetime of reactive components of the POC (*a*), and the shape of the distribution of their reactivities (ν). Our choice of *a* and ν are guided by a global parameter compilation (Arndt et al., 2013; Freitas et al., 2021) that reflects typically observed RCM parameter variability across the various depositional environments considered here, as well as existing model applications that are validated using a large body of interdisciplinary data. This representation of POC degradation produces heterotrophic reaction rates that vary by eight orders of magnitude across different marine environments, and is appropriate for broad regions of the seafloor. The presented approach (as is the case for all model predictions in general) is subject to uncertainties and therefore future work in refining our set of parameters will serve to advance predictive capabilities further.

Our modeling framework divides marine sediments into three major catabolic zones in which POC degradation is coupled to O_2_ reduction (oxic zone), sulfate-reduction and methanogenesis (Canfield et al., 2005; Jørgensen and Kasten, 2006; Thullner et al., 2009). We define the geographic boundaries of the reaction networks according to combined global datasets of pore water concentrations in marine sediments compiled and summarized in Glud et al. (1998), Glud (2008), Egger et al. (2018) and Bradley et al. (2020) and extrapolations of them (D’Hondt et al., 2015; Egger et al., 2018). This accounts for broad patterns in major catabolic zones but neglects other known catabolic pathways such as nitrate, iron, and manganese-reduction, fermentation, and secondary redox processes such as chemolithotrophic reactions like Fe^2+^ oxidation, which may all directly or indirectly supply energy and control global sediment redox conditions. Previous work has shown that the other electron acceptors such as nitrate and metal oxides are largely unimportant in global POC degradation (Middelburg and Levin, 2009; Thullner et al., 2009; Arndt et al., 2013; Dale et al., 2015) – mainly due to their low concentration compared to sulfate, the additional consumption of Fe- and Mn-oxides in a range of rapid secondary redox reactions, and the rapid exchange of oxygen in shallow sediment. Energy may also be provided by fermentation, although its importance is not well known, and nor is the Gibbs energy realized by fermentation – since it is quite variable and depends sharply on the exact fermentation reaction and the environmental conditions under which the reaction takes place (LaRowe and Amend, 2019). Despite the likely misattribution of some amount of energy between functional groups that are included and omitted from our study, the overall effect on the resulting average power used per cell is likely to not be significant, due to the order-of-magnitude differences in energy and POC fluxes across space and time. We use acetate (CH_3_COO^–^) as a proxy for POC in our Gibbs energy calculations. We expect fractional changes in Gibbs energies associated with the choice of organic compound, but this does not meaningfully affect our results [see calculations reported in Bradley et al. (2020)]. We also expect a certain amount of variability in the Gibbs energy of the major geochemical reactions considered in this study to arise from spatial heterogeneities in the concentrations of reactants and products, as well as temperature and pressure (LaRowe et al., 2017). However, uncertainties in the Gibbs energies of each reaction do not meaningfully affect our power calculations, considering that microbial power utilization spans a range of five orders of magnitude, and the deviation of Gibbs energies from our nominal values is minor [see calculations reported in Bradley et al. (2020)].

## Conclusion

We find that trends in cell abundance, POC storage and degradation, and microbial power utilization are mainly structured by depositional setting and redox conditions, rather than sediment depth and age. Sediments deposited on continental shelves and margins are predominantly anoxic and contain active microbial cells that decline in power utilization in deeper and older settings. Conversely, microorganisms in abyssal sediments use consistently low amounts of power across large gradients in sediment depth and age. Overall, we demonstrate broad global-scale connections between the depositional setting and activity of deep biosphere microorganisms.

## Data Availability Statement

The data related to this study are available at https://www.bco-dmo.org/project/776336.

## Author Contributions

JB designed the study with contributions from DL and JA, performed the additional calculations, analyzed the data, and wrote the manuscript with significant contributions from all co-authors. DL, JB, SA, and EB-G designed and implemented the RTM. All authors contributed to the article and approved the submitted version.

## Conflict of Interest

The authors declare that the research was conducted in the absence of any commercial or financial relationships that could be construed as a potential conflict of interest.

## Publisher’s Note

All claims expressed in this article are solely those of the authors and do not necessarily represent those of their affiliated organizations, or those of the publisher, the editors and the reviewers. Any product that may be evaluated in this article, or claim that may be made by its manufacturer, is not guaranteed or endorsed by the publisher.
