# Supplementary material for: Sources and Fluxes of Organic Carbon and Energy to Microorganisms in Global Marine Sediments
Source: Front Microbiol. 2022 Jul 7;13:910694. doi: 10.3389/fmicb.2022.910694 (PMC9301249; doi:10.3389/fmicb.2022.910694)
Supplement: Supplementary file 1 [file Image_1.pdf]

Sources and fluxes of organic carbon and energy to microorganisms in global marine sediments.

James A. Bradley[1,2]*, Sandra Arndt[3], Jan P. Amend[4], Ewa Burwicz-Galerne[5], Douglas E. LaRowe[4]

[1] Queen Mary University of London, London, UK
[2] GFZ German Research Center for Geosciences
[3] Université Libre de Bruxelles, Brussels, Belgium
[4] University of Southern California, Los Angeles, CA, USA
[5] MARUM Center for Marine Environmental Sciences, Faculty of Geosciences, University of Bremen, Bremen, Germany.

1. Supplementary Methods

1.1 Reaction transport model

Following the approaches described in (Bradley et al., 2020; Dale et al., 2015; LaRowe et al., 2020), we use a multi-G approximation of the RCM for the bioturbated sediment. Within the bioturbated zone, POC is represented by 500 distinct fractions that are degraded according to a first-order organic matter degradation rate law with a degradation rate constant, k_i:

$$R_{POC} = \sum_{i=1}^{500} k_i \cdot POC_i(z)$$

(S1)

Where

$$POC_i(0) = F_i \cdot POC$$

(S2)

The initial proportion of total organic matter in fraction i, F_i, as well as its respective reactivity, k_i, can be determined through the initial probability density function that determines the concentration of organic matter having a degradability between k and $k+dk$ at time 0 (Eq. 4). The initial fraction of total POC characterized by a distinct reactivity k is given by:

$$f(k,0) = \frac{om(k,0)}{POC_0} = \frac{ia^{\nu} \cdot k^{\nu-1} \cdot e^{-a \cdot k}}{\Gamma(\nu)}$$

(S3)

The initial fraction of POC within the reactivity range between 0 and k, i.e. having a reactivity $\leq k$, is then given by integrating Eq. (S3), assuming $a, \nu, k > 0$:

$$F(k,0) = \int_0^k f(0,k)dk$$
$$= \int_0^k \frac{a^{\nu} \cdot k^{\nu-1} \cdot e^{-a \cdot k}}{\Gamma(\nu)} dk = \frac{a^{\nu} \cdot k^{\nu} \cdot (a \cdot k)^{-\nu}(\Gamma(\nu) - \Gamma(\nu, a \cdot k))}{\Gamma(\nu)}$$
$$= \left(\frac{1 - \Gamma(\nu, a \cdot k)}{\Gamma(\nu)}\right)$$

(S4)

Where $\Gamma(v,a\cdot k)$ denotes the inverse gamma function.

In the bioturbated sediment, the RCM is approximated by dividing the reactivity range k=[10^{-15}, $10^{(-\log(a)+2)}$] into 500 equal reactivity bins, k_j, thus ensuring a comprehensive approximation of the gamma function defined by the respective a and v values. The initial fraction, F_i, of total POC within the reactivity bin k_{j-1} and k_j (and thus with reactivity $k_i = k_{j-1} + (k_j - k_{j-1})/2$) in the 500G model can then be calculated as:

$$F_i = F(k_j, 0) - F(k_{j-1}, 0) \tag{S5}$$

The most reactive fraction, F_{500}, with reactivity $k_{500}=10^{-\log(a)+2}$ year^{-1} was calculated on the basis of the upper incomplete gamma function:

$$F_{500} = \int_{k_{500}}^{\infty} f(k_{500}, 0)dk = \frac{\Gamma(v, a \cdot k_{500})}{\Gamma(v)} \tag{S6}$$

The derived rate constants were then used in Eq. (2) by expressing R_{POC} according to Eq. (S1) to determine POC concentrations and degradation rates in the Holocene bioturbated layer (<10 cm). Assuming steady-state conditions, the general solution of Eq. (2) for each organic matter fraction i is given by:

$$POC_i(z) = A_i e^{(\alpha_i z)} + B_i e^{(b_i z)} \tag{S7}$$

Where

$$\alpha_i = \frac{\omega - \sqrt{\omega^2 + 4D_b \cdot k_i}}{2D_b} \tag{S8}$$

$$b_i = \frac{\omega + \sqrt{\omega^2 + 4D_b \cdot k_i}}{2D_b} \tag{S9}$$

And

$$POC(z) = \sum_{i=1}^{500} POC_i(z) \tag{S10}$$

The integration constants A_i and B_i are defined by the chosen boundary conditions. Here, we apply a known POC concentration at the sediment-water interface ($POC(0) = POC_0$) and assume continuity (equal flux and concentration) across the bottom of the bioturbated layer, i.e.:

$$POC(zbio) = POC_{zbio}\ ; \left.\frac{-D_b dPOC}{dz}\right|_{zbio} = 0 \tag{S11}$$

Below the Holocene bioturbated zone, the values of $age(z)$ that are required to evaluate Eq. (6) are calculated using burial rates, ω(z), porosity depth profiles, Φ(z), and the apparent age of organic matter at the lower limit of the Holocene bioturbated zone, age_{zbio}. By inserting POC_{bio} and age_{zbio} into Eq. (6) for $POC(z)$ and $age(z)$, respectively, age_{zbio} can be solved:

$$age_{zbio} = \frac{-a \cdot (\exp\,(\ln\,(POC_{bio}/POC_0)/v) - 1)}{\exp\,(\ln\,(POC_{bio}/POC_0)/v)}$$

(S12)

Assuming an exponentially decreasing porosity, Eq. (2), and steady-state compaction, the burial velocity, ω at depth z is then (e.g. (Berner, 1980)):

$$\omega(z) = \left(\frac{1-\Phi_0}{1-\Phi(z)}\right)\omega_0$$

(S13)

Where ω_0 corresponds to the burial velocity at the SWI. The age of a given sediment layer at depth z below the Holocene bioturbated zone, *age(z)*, is given by (e.g., (Berner, 1980)):

$$age(z) = \int_0^z \omega^{-1}\, dz$$

(S14)

Substituting Eq. (S13) into Eq. (S14) results in:

$$age(z) = \frac{1}{(1-\Phi_0)\omega_0} \int_0^z (1-\Phi)\, dz$$

(S15)

Which, upon integration, leads to:

$$age(z) = \frac{z + \frac{\Phi_0}{c_0}\cdot(exp(-c_0\cdot z)-1)}{\omega_0\cdot(1-\Phi_0)}$$

(S16)

The age of POC below the Holocene bioturbated zone is thus given by:

$$age(z) = age_{zbio} + \frac{z + \frac{\Phi_0}{c_0}\cdot\left(exp\left(-c_0\cdot(z-z_{bio})\right)-1\right)}{\omega_0\cdot(1-\Phi_0)}$$

(S17)

The depth distribution of organic matter in marine sediments deposited since the beginning of the Quaternary Period can thus be calculated with knowledge of the sedimentation rate, level of bioturbation, porosity structure, bulk organic matter concentration at the SWI and the distribution of organic compounds across the reactivity range at the sediment-water interface.

1.2 Total POC budget

The fraction of total POC preserved in a layer of sediment that accumulated over a given time interval, $\overline{PE_i}$, is given as the ratio of the total amount of POC stored in the *i*th sediment layer (*i*=Holocene bioturbated (0 to z_{bio}), Holocene non-bioturbated (z_{bio} to z_{holo}), Pleistocene (z_{holo} to z_{pleis})), $\overline{POC_i}$ (g C cm^{-2}) and the total steady-state input of POC to that respective layer, $\overline{I_i}$, (g C cm^{-2}):

$$\overline{PE_i} = \overline{POC_i}/\overline{I_i}$$

(S18)

Where

$$\overline{POC_i} = \int_{z_i}^{z_{i-1}} POC(z)dz$$

(S19)

And

$$\overline{I_i} = POC(z_i - 1) \cdot \Delta z_i \tag{S20}$$

The amount of POC degraded in the *i*th layer, $\overline{R_i}$, is given by:

$$\overline{R_i} = \int_{z_i}^{z_{i-1}} k(z) \cdot POC(z) dz \tag{S21}$$

Two different global datasets (0.25°×0.25°) of values for ω, the sedimentation rate, are used: one for Holocene bioturbated and Holocene sediments and the other for Pleistocene sediments.

S1.1 POC reaction transport modelling					
POC at the sediment water interface	Established in (Bradley et al., 2020; LaRowe et al., 2020) using data from (Romankevich et al., 2009; Seiter et al., 2004; Wallmann et al., 2012)				
Sedimentation rate	Established in (Bradley et al., 2020; LaRowe et al., 2020) using data from (Burwicz et al., 2011).				
Bioturbation coefficient	Established in (Bradley et al., 2020; LaRowe et al., 2020) using data from (Middelburg et al., 1997)				
Parameter	Definition	Shelf	Margin	Abyss	Units
Φ_0	Sediment porosity at the sediment-water interface [a]	0.45 [a]	0.74 [a]	0.70 [a]	
c_0	Sediment compaction length scale [b]	0.50×10^{-3} [b]	1.70×10^{-4} [b,c]	0.85×10^{-3} [b]	m^{-1}
a	Reactive continuum age parameter	$10^{(3.35-14.81\cdot\omega)}$ [d]	$10^{(3.35-14.81\cdot\omega)}$ [d]	$10^{(3.35-14.81\cdot\omega)}$ [d]	years
v	Reactive continuum distribution parameter	0.125	0.125	0.125	

S1.2 Global reaction network, electron acceptors and catabolic zones	
Oxygen penetration depth	Established in (Bradley et al., 2020) using data from (Black et al., 2001; Canfield et al., 1993; D'Hondt et al., 2015; Giordani et al., 2002; Glud et al., 1994, 1998, 1999, 2003, 2009; Jahnke et al., 1989; Lansard et al., 2008, 2009; Sachs et al., 2009; Wenzhöfer et al., 2001b, 2001a; Wenzhöfer and Glud, 2002; Witte et al., 2003b, 2003a) and elsewhere as described in (Bradley et al., 2020).
Sulfate-methane transition zone	Established in (Bradley et al., 2020) using data from (Egger et al., 2018).
Redox conditions	Established in (Bradley et al., 2020) using data from (D'Hondt et al., 2004, 2015; Jørgensen and Parkes, 2010).

S1.3 Cell abundance	
Cell abundance	Established in (Bradley et al., 2020) using modelling from (Kallmeyer et al., 2012)

Supplementary Table S1. Summary of major datasets used to inform the modelling simulations and parameter values. [a] (LaRowe et al., 2017); [b] These values are representative of a sandstone-siltstone mixture (shelf), a sandstone-siltstone- shale combination (margin) and typical shales and biogenic-dominated sediments (abyss) (Hantschel and Kauerauf, 2009); [c] (Wallmann et al., 2012); [d] based on global compilation by (Arndt et al., 2013), ω represents sedimentation rate (cm yr^{-1}).

Reaction no.	Metabolism	Reaction
1	Aerobic heterotrophy	$C_2H_3O_2^- + 2O_2 \rightleftharpoons H^+ + 2HCO_3^-$
2	Sulfate reduction	$C_2H_3O_2^- + SO_4^{2-} \rightleftharpoons HS^- + 2HCO_3^-$
3	Methanogenesis	$C_2H_3O_2^- + H_2O \rightleftharpoons CH_4 + HCO_3^-$

Supplementary Table S2. Catabolic pathways for POC oxidation considered in this study.

Metabolism	ΔG_r	$C_2H_3O_2^-$	O_2	HCO_3^-	SO_4^{2-}	HS^-	CH_4	pH
Aerobic heterotrophy	-34.7200	0.00063[a,b]	0.00008[c]	0.0023[c]				7.78[c]
Sulfate reduction	-2.6833	0.00063[a,b]		0.0250[d]	0.0154[e]	1×10^{-6} [d]		
Methanogenesis	-0.9872	0.00063[a,b]		0.0280[d]			0.00060[d]	

Supplementary Table S3. Gibbs energies and concentrations of reactants and products used to calculate the Gibbs energies for each catabolic reaction. Gibbs energies are provided in kJ g^{-1} C. Concentrations are provided in moles (M). Concentrations for the various scenarios are taken from the literature and are intended to represent the variation that is present in typical sedimentary settings and not extreme values that are not globally representative. [a] (Burdige and Gardner, 1998); [b] (Fox et al., 2018); [c] (D'Hondt et al., 2015); [d] (Jørgensen and Parkes, 2010); [e] (D'Hondt et al., 2004).

Supplementary References

Arndt, S., Jørgensen, B. B., LaRowe, D. E., Middelburg, J. J., Pancost, R. D., and Regnier, P. (2013). Quantifying the degradation of organic matter in marine sediments: A review and synthesis. *Earth-Science Rev.* 123, 53–86. doi:DOI 10.1016/j.earscirev2013.02.008.

Berner, R. A. (1980). *Early Diagenesis: A Theoretical Approach*. Available at: http://books.google.com/books?id=weRFglCVBkUC&pgis=1.

Black, K. S., Fones, G. R., Peppe, O. C., Kennedy, H. A., and Bentaleb, I. (2001). An autonomous benthic lander:: preliminary observations from the UK BENBO thematic programme. *Cont. Shelf Res.* 21, 859–877. doi:https://doi.org/10.1016/S0278-4343(00)00116-3.

Bradley, J. A., Arndt, S., Amend, J. P., Burwicz, E., Dale, A. W., Egger, M., et al. (2020). Widespread energy limitation to life in global subseafloor sediments. *Sci. Adv.* 6, eaba0697. doi:10.1126/sciadv.aba0697.

Burdige, D. J., and Gardner, K. G. (1998). Molecular weight distribution of dissolved organic carbon in marine sediment pore waters. *Mar. Chem.* 62, 45–64. doi:https://doi.org/10.1016/S0304-4203(98)00035-8.

Burwicz, E. B., Rüpke, L. H., and Wallmann, K. (2011). Estimation of the global amount of submarine gas hydrates formed via microbial methane formation based on numerical reaction-transport modeling and a novel parameterization of Holocene sedimentation. *Geochim. Cosmochim. Acta* 75, 4562–4576. doi:10.1016/j.gca.2011.05.029.

Canfield, D. E., Jørgensen, B. B., Fossing, H., Glud, R., Gundersen, J., Ramsing, N. B., et al. (1993). Pathways of organic carbon oxidation in three continental margin sediments. *Mar. Geol.* 113, 27–40. doi:https://doi.org/10.1016/0025-3227(93)90147-N.

D'Hondt, S., Inagaki, F., Zarikian, C. A., Abrams, L. J., Dubois, N., Engelhardt, T., et al. (2015). Presence of oxygen and aerobic communities from sea floor to basement in deep-sea sediments. *Nat. Geosci.* 8, 299–304. doi:10.1038/ngeo2387.

D'Hondt, S., Jørgensen, B. B., Miller, D. J., Batzke, A., Blake, R., Cragg, B. A., et al. (2004). Distributions of microbial activities in deep subseafloor sediments. *Science (80-.).* 306, 2216–2221. doi:10.1126/science.1101155.

Dale, A. W., Nickelsen, L., Scholz, F., Hensen, C., Oschlies, A., and Wallmann, K. (2015). A revised global estimate of dissolved iron fluxes from marine sediments. *Global Biogeochem. Cycles* 29, 691–707. doi:10.1002/2014GB005017.

Egger, M., Riedinger, N., Mogollón, J. M., and Jørgensen, B. B. (2018). Global diffusive fluxes of methane in marine sediments. *Nat. Geosci.* 11, 421–425. doi:10.1038/s41561-018-0122-8.

Fox, C. A., Abdulla, H. A., Burdige, D. J., Lewicki, J. P., and Komada, T. (2018). Composition of Dissolved Organic Matter in Pore Waters of Anoxic Marine Sediments Analyzed by 1H Nuclear Magnetic Resonance Spectroscopy . *Front. Mar. Sci.* 5, 172. Available at: https://www.frontiersin.org/article/10.3389/fmars.2018.00172.

Giordani, P., Helder, W., Koning, E., Miserocchi, S., Danovaro, R., and Malaguti, A. (2002). Gradients of benthic–pelagic coupling and carbon budgets in the Adriatic and Northern Ionian Sea. *J. Mar. Syst.* 33–34, 365–387. doi:https://doi.org/10.1016/S0924-7963(02)00067-2.

Glud, R., Gundersen, J. K., Barker Jørgensen, B., Revsbech, N. P., and Schulz, H. D. (1994). Diffusive and total oxygen uptake of deep-sea sediments in the eastern South Atlantic Ocean:in situ and laboratory measurements. *Deep Sea Res. Part I Oceanogr. Res. Pap.* 41, 1767–1788. doi:https://doi.org/10.1016/0967-0637(94)90072-8.

Glud, R., Holby, O., Hoffmann, F., and Canfield, D. (1998). Benthic mineralization and exchange in Arctic sediments (Svalbard, Norway). *Mar. Ecol. Prog. Ser.* 173, 237–251.

doi:10.3354/meps173237.
Glud, R. N., Gundersen, J. K., and Holby, O. (1999). Benthic in situ respiration in the upwelling area off central Chile. *Mar. Ecol. Prog. Ser.* 186, 9–18. Available at: http://www.jstor.org/stable/24853288.
Glud, R. N., Gundersen, J. K., Røy, H., and Jørgensen, B. B. (2003). Seasonal dynamics of benthic O2 uptake in a semienclosed bay: Importance of diffusion and faunal activity. *Limnol. Oceanogr.* 48, 1265–1276. doi:10.4319/lo.2003.48.3.1265.
Glud, R. N., Stahl, H., Berg, P., Wenzhöfer, F., Oguri, K., and Kitazato, H. (2009). In situ microscale variation in distribution and consumption of 2: A case study from a deep ocean margin sediment (Sagami Bay, Japan). *Limnol. Oceanogr.* 54, 1–12. doi:10.4319/lo.2009.54.1.0001.
Hantschel, T., and Kauerauf, A. I. (2009). *Fundamentals of Basin and Petroleum Systems Modeling*. Berlin: Springer-Verlag.
Jahnke, R. A., Emerson, S. R., Reimers, C. E., Schuffert, J., Ruttenberg, K., and Archer, D. (1989). Benthic recycling of biogenic debris in the eastern tropical Atlantic Ocean. *Geochim. Cosmochim. Acta* 53, 2947–2960. doi:https://doi.org/10.1016/0016-7037(89)90171-3.
Jørgensen, B. B., and Parkes, R. J. (2010). Role of sulfate reduction and methane production by organic carbon degradation in eutrophic fjord sediments (Limfjorden, Denmark). *Limnol. Oceanogr.* 55, 1338–1352. doi:10.4319/lo.2010.55.3.1338.
Kallmeyer, J., Pockalny, R., Adhikari, R. R., Smith, D. C., and D'Hondt, S. (2012). Global distribution of microbial abundance and biomass in subseafloor sediment. *Proc. Natl. Acad. Sci.* 109, 16213–16216. doi:10.1073/pnas.1203849109.
Lansard, B., Rabouille, C., Denis, L., and Grenz, C. (2008). In situ oxygen uptake rates by coastal sediments under the influence of the Rhône River (NW Mediterranean Sea). *Cont. Shelf Res.* 28, 1501–1510. doi:https://doi.org/10.1016/j.csr.2007.10.010.
Lansard, B., Rabouille, C., Denis, L., and Grenz, C. (2009). Benthic remineralization at the land–ocean interface: A case study of the Rhône River (NW Mediterranean Sea). *Estuar. Coast. Shelf Sci.* 81, 544–554. doi:https://doi.org/10.1016/j.ecss.2008.11.025.
LaRowe, D. E., Arndt, S., Bradley, J. A., Burwicz, E., Dale, A., and Amend, J. P. (2020). Organic carbon and microbial activity in marine sediments on a global scale throughout the Quaternary. *Geochim. Cosmochim. Acta* 286, 227–247. doi:10.1016/j.gca.2020.07.017.
LaRowe, D. E., Burwicz, E., Arndt, S., Dale, A. W., and Amend, J. P. (2017). Temperature and volume of global marine sediments. *Geol.* . doi:10.1130/G38601.1.
Middelburg, J. J., Soetaert, K., and Herman, P. M. J. (1997). Empirical relationships for use in global diagenetic models. *Deep Sea Res. Part I Oceanogr. Res. Pap.* 44, 327–344. doi:https://doi.org/10.1016/S0967-0637(96)00101-X.
Romankevich, E. A., Vetrov, A. A., and Peresypkin, V. I. (2009). Organic matter of the World Ocean. *Russ. Geol. Geophys.* 50, 299–307. doi:https://doi.org/10.1016/j.rgg.2009.03.013.
Sachs, O., Sauter, E. J., Schlüter, M., Rutgers van der Loeff, M. M., Jerosch, K., and Holby, O. (2009). Benthic organic carbon flux and oxygen penetration reflect different plankton provinces in the Southern Ocean. *Deep Sea Res. Part I Oceanogr. Res. Pap.* 56, 1319–1335. doi:https://doi.org/10.1016/j.dsr.2009.02.003.
Seiter, K., Hensen, C., Schröter, J., and Zabel, M. (2004). Organic carbon content in surface sediments—defining regional provinces. *Deep Sea Res. Part I Oceanogr. Res. Pap.* 51, 2001–2026. doi:https://doi.org/10.1016/j.dsr.2004.06.014.
Wallmann, K., Pinero, E., Burwicz, E., Haeckel, M., Hensen, C., Dale, A., et al. (2012). The Global Inventory of Methane Hydrate in Marine Sediments: A Theoretical Approach.

Energies 5. doi:10.3390/en5072449.
Wenzhöfer, F., Adler, M., Kohls, O., Hensen, C., Strotmann, B., Boehme, S., et al. (2001a). Calcite dissolution driven by benthic mineralization in the deep-sea: in situ measurements of Ca2+, pH, pCO2 and O2. *Geochim. Cosmochim. Acta* 65, 2677–2690. doi:https://doi.org/10.1016/S0016-7037(01)00620-2.
Wenzhöfer, F., and Glud, R. N. (2002). Benthic carbon mineralization in the Atlantic: a synthesis based on in situ data from the last decade. *Deep Sea Res. Part I Oceanogr. Res. Pap.* 49, 1255–1279. doi:https://doi.org/10.1016/S0967-0637(02)00025-0.
Wenzhöfer, F., Holby, O., and Kohls, O. (2001b). Deep penetrating benthic oxygen profiles measured in situ by oxygen optodes. *Deep Sea Res. Part I Oceanogr. Res. Pap.* 48, 1741–1755. doi:https://doi.org/10.1016/S0967-0637(00)00108-4.
Witte, U., Aberle, N., Sand, M., and Wenzhöfer, F. (2003a). Rapid response of a deep-sea benthic community to POM enrichment: *Mar. Ecol. Prog. Ser.* 251, 27–36. Available at: http://www.jstor.org/stable/24866553.
Witte, U., Wenzhöfer, F., Sommer, S., Boetius, A., Heinz, P., Aberle, N., et al. (2003b). In situ experimental evidence of the fate of a phytodetritus pulse at the abyssal sea floor. *Nature* 424, 763. Available at: https://doi.org/10.1038/nature01799.
